# Antibacterial and Antiviral Properties of Tetrahydrocurcumin-Based Formulations: An Overview of Their Metabolism in Different Microbiotic Compartments

**DOI:** 10.3390/life12111708

**Published:** 2022-10-26

**Authors:** Natalija Atanasova-Panchevska, Radoslav Stojchevski, Nikola Hadzi-Petrushev, Vadim Mitrokhin, Dimiter Avtanski, Mitko Mladenov

**Affiliations:** 1 Faculty of Natural Sciences and Mathematics, Institute of Biology, Ss. Cyril and Methodius University, P.O. Box 162, 1000 Skopje, North Macedonia; 2 Friedman Diabetes Institute, Lenox Hill Hospital, Northwell Health, 110 E 59th Street, New York, NY 10022, USA; 3 Department of Physiology, Pirogov Russian National Research Medical University, Ostrovityanova Street, 1, 117997 Moscow, Russia

**Keywords:** tetrahydrocurcumin, intestinal microbiota, antibacterial properties, antiviral properties, curcuminoid formulation, animal models

## Abstract

In this review, the basic metabolic characteristics of the curcuminoid tetrahydrocurcumin (THC) at the level of the intestinal microbiota were addressed. Special attention was given to the bactericidal effects of one of the THC-phospholipid formulations, which has shown greater bioavailability and activity than pure THC. Similarly, quinoline derivatives and amino acid conjugates of THC have also shown antibacterial effects in the gut. The microbial effect of pure THC is particularly pronounced in pathophysiological conditions related to the function of the intestinal microbiota, such as type II diabetes. Furthermore, the antiviral characteristics of Cur compared to those of THC are more pronounced in preventing the influenza virus. In the case of HIV infections, the new microemulsion gel formulations of THC possess high retention during preventive application in the vagina and, at the same time, do not disturb the vaginal microbiota, which is critical in maintaining low vaginal pH. Based on the reviewed literature, finding new formulations of THC which can increase its bioavailability and activity and emphasize its antibacterial and antiviral characteristics could be very important. Applying such THC formulations in preventing and treating ailments related to the microbiotic compartments in the body would be beneficial from a medical point of view.

## 1. Background

Curcumin (Cur) has occupied scientific interest in the last decade [1] due to its possible bioactivity in humans [2,3]. On the other hand, Cur has very poor oral absorption, insignificant biodistribution, and low systemic bioavailability. To increase Cur bioavailability, several methods such as phytosome, liposome, and micelle formulation, as well as Cur co-formulation with adjuvants such as piperine, have been utilized [4]. Although new formulations improve curcuminoid absorption in the small intestine, a significant proportion of curcuminoids reach the colon and are excreted [5]. This is an essential step since it has been demonstrated that the animal gut microbiota conducts several metabolic/catabolic reactions to curcuminoids, which should be included in the overall assessment of Cur bioactivity and bioavailability [6,7].

Tetrahydrocurcumin (THC), a reduced analog of Cur with phenolic and -diketo moieties [8], has been identified as an active Cur metabolite in the gastrointestinal system. THC has been demonstrated to be superior to Cur in terms of anti-diabetic [9], anti-hyperlipidemic, anti-oxidant, and anti-cancerogenic effects which may be related to the biotransformation of Cur by intestinal microbiota [10,11]. Despite this advantage, there have been several efforts to develop new, even better THC formulations in order to increase the work of the gut flora. Different studies on THCs’ intestinal bioconversion are being conducted in order to reveal the possible pool of catabolites generated from various THC formulations via gut microbiota-driven metabolism [8,9,10]. In this direction, Duan et al. [12] reported antitumor activity of three new THC derivatives, but since the anti-cancerogenic efficacy of THC derivatives is not the objective of this study, regarding their mechanisms of action the reader is directed to the relevant works. More relevantly, the results of Ou et al. [13] have a very important contribution, implying that THC might potentially target specific components of the viral replication machinery or block cellular signaling pathways required for viral reproduction. In light of this, THC was proposed as an alternative to Cur in the prevention of human immunodeficiency virus (HIV) infection. Moreover, the recently employed computer-assisted in silico molecular docking and molecular dynamic simulations, focused on the comparison between the gp120-Cur and gp120-THC inhibitory effects, demonstrate that the THC-gp120 complex has greater chemical stability. This is another example of improved antiviral properties based on increased upregulation as a result of the changed formulation of the THC complex [14].

Henceforth, the current study attempted to review the known interactions and the data about the intestinal metabolism of various THC formulations, as well as to appropriately identify clinically or in silico tested THC formulations based on their antibacterial and antiviral capabilities.

## 2. Effect of the Curcuminoid Formulation on Its Metabolism in the Intestinal Microbiota

**Figure 1:**
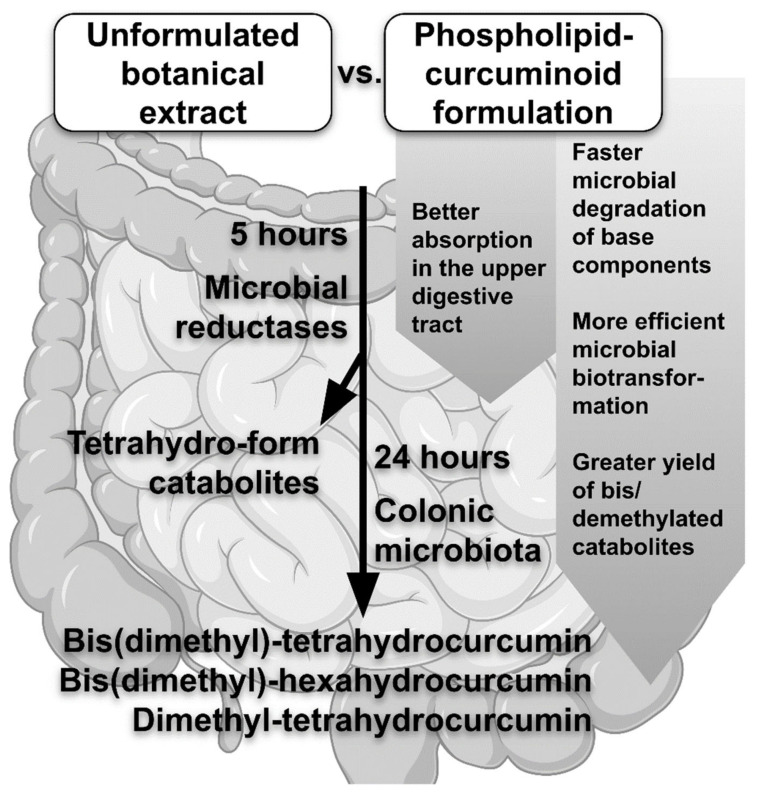
Effects of the curcuminoid formulation on its metabolism in the intestinal microbiota.

The study of Bresciani et al. [15] added a new value to our understanding of the metabolism of human intestinal bacteria, especially in terms of the metabolic fate of different formulations of curcuminoids. Concerning qualitative differences, no variance has been found between the lecithin-curcuminoid formulation and the unformulated botanical extract, albeit the phospholipid formulation undergoes faster microbial degradation of the base components compared to the unformulated Cur extract [15]. The curcuminoids in the phospholipid delivery method undergo more efficient microbial biotransformation than the Cur extract alone. The comparison of lecithin to a basic botanical extract formulation also revealed that the primary difference was in the synthesis of metabolites after 24 h of microbial incubation [15]. Curcuminoid catabolites are mostly recovered as tetrahydro-forms after 5 h of incubation due to the activity of microbial reductases. The parent curcuminoids are mainly digested by the colonic microbiota within 24 hours, leading to demethylation and/or bis(demethylation). The main curcuminoid catabolites formed after 24 h of microbial fermentation are bis(dimethyl)-tetrahydrocurcumin (BDM-THC), bis(dimethyl)-hexahydrocurcumin (BDM-HHC), and dimethyl-tetrahydrocurcumin (DM-THC), and the yield of these compounds is substantially greater in case of the phospholipid formulation [15]. To the best of our knowledge, Bresciani et al. [15] were the first to report that BDM-HHC is one of the basic curcuminoids extracted from microbial catabolites. These findings demonstrate that the human colonic microbiota is capable of generating demethylated curcuminoids [6]. The curcuminoids administered in a phospholipid formulation are also better absorbed in the upper digestive tract compared to the curcuminoids administered in their parent, unformulated form [16]. However, the formulation’s effect on microbial catabolism of curcuminoids in this part of the gut is unknown. The novel findings in Bresciani et al. [15] study support the importance of the intestinal microbiota in curcuminoid degradation, which was also already shown in the case of other polyphenols [17]. Furthermore, compared to the unformulated extract, the phospholipid formulation leads to a more effective microbial biotransformation of Cur [17] (Figure 1).

From all the above, the in vivo bioavailability and the potential bioactivity attributed to curcuminoids should be reassessed. The phospholipid formulation by combining different curcuminoids might create a composition with considerably increased bioavailability, activity, and microbial stability.

## 3. Quinoline Derivatives and AA Conjugates of THC and Their Antibacterial Properties

Manjunatha et al. [18] investigated the antibacterial activity of quinoline derivatives of THC (Figure 1) against two Gram-positive (*B. cereus* and *S. aureus*) and two Gram-negative bacteria (*E. coli* and *Y. enterocolitica*).

Table 1 presents the data based on the minimum inhibitory concentration (MIC) values of quinoline derivatives of THC in the form of compounds from 1a to 1f, published by the same authors. Among all examined derivatives, 1c is proven to be the most efficient against Gram-negative bacteria, with the lowest MIC values against the tested strains. Compounds 1c and 1b were shown to be highly efficient against all bacteria tested in this study, whereas compound 1e was found to be relatively effective [18]. Furthermore, 1e and 1f were more active against *B. cereus* than against other bacteria [18].

**Figure 2:**
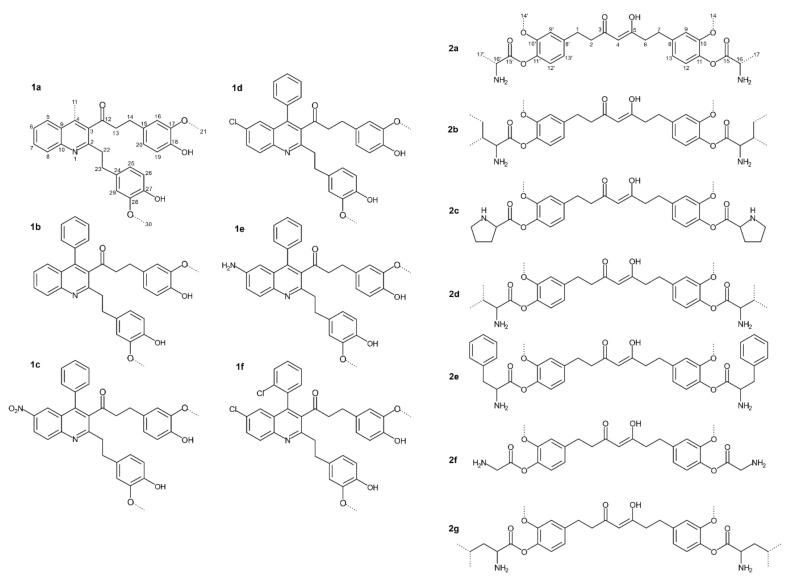
Quinoline derivatives and amino acid conjugates of THC: **1a**.—1-(2-(4-hydroxy-3-methoxyphenethyl)-4-methylquinolin-3-yl)-3-(4-hydroxy-3-methoxyphenyl)propan-1-one; **1b**.—1-(2-(4-Hydroxy-3-methoxyphenethyl)-4-phenylquinolin-3-yl)-3-(4-hydroxy-3-methoxyphenyl) propan-1-one; **1c**.—1-(2-(4-Hydroxy-3-methoxyphenethyl)-6-nitro-4-phenylquinolin-3-yl)-3-(4-hydroxy-3-methoxyphenyl)propan-1-one; **1d**.—1-(6-Chloro-2-(4-hydroxy-3-methoxyphenethyl)-4-phenylquinolin-3-yl)-3-(4-hydroxy-3-methoxyphenyl)propan-1-one; **1e**.—1-(6-Amino-2-(4-hydroxy-3-methoxyphenethyl)-4-phenylquinolin-3-yl)-3-(4-hydroxy-3-methoxyphenyl)propan-1-one; **1f**.—1-(6-Chloro-4-(2-chlorophenyl)-2-(4-hydroxy-3-methoxypheneth-yl) quinolin-3-yl)-3-(4-hydroxy-3-methoxyphenyl) propan-1-one; **2a**. Alanine. **2b**. Isoleucine. **2c**. Proline. **2d**. Valine. **2e**. Phenylalanine. **2f**. Glycine. **2g**. Leucine. (Modified from Manjunatha et al. [18,19].

The same group also tested amino acid (AA) conjugates of THC (presented in Figure 2a–g) and reported antibacterial activity against two Gram-positive (*B. cereus* and *S. aureus*) and two Gram-negative (*E. coli* and *Y. enterocolitica*) species [19].

Table 1 also presents the MIC-related data for the AA conjugates of THC, indicating that all tested conjugates in general appear to be more active compared to the quinoline derivatives. Additionally, both the AA conjugates and the quinoline derivatives were also substantially more active than THC [18,19] (MIC data for THC not shown).

At the same time, THC-glycine and THC-valine conjugates (2f and 2d, respectively) are the most effective against *B. cereus*. According to Manjunatha et al. [19], THC and its AA conjugates suppress bacterial growth in the following order of strength: 2f > 2d > 2a > 2c > 2b > 2e > 2g > THC. Actually, the authors have shown that compound 2f inhibited the growth of *S. aureus* the most, whereas compound 2g led to minimal inhibition, following the growth inhibition trend: 2f > 2c > 2d > 2a = 2b = 2e > THC > 2g. The same research has confirmed that 2f mostly inhibited the growth of *E. coli* and *Y. enterocolitica*, and their MIC values were associated with the trend of inhibition (Table 1).

Kapoor et al. [20] have also found that some Cur bio-conjugates comprising esters and peptides have stronger antifungal and antibacterial properties, which can be related to improved cell uptake, increased cellular concentration, and improved receptor binding capacity. The AA part of the derivatives appears to make the THC conjugates hydrophilic, which aids in the increased absorption of the covalently bound THC into bacterial cells.

A similar pattern has been discovered for various natural and synthetic compounds, which exhibit varying activity based on the derivative structure and the studied bacterial strain [21,22]. Comparing the activity of various THC derivatives against bacteria demonstrated that virtually all of the investigated THC derivatives (Figure 2) have higher MIC values against Gram-negative than Gram-positive bacteria, as this impact is primarily due to differences in the compound structure [23,24]. Gram-positive bacteria have an exterior peptidoglycan layer that acts as a permeability barrier [25]. The outer phospholipid membrane of Gram-negative bacteria is impervious to lipophilic solutes [26]. Furthermore, porins in the cell membrane serve as a selective barrier to hydrophilic solutes, rendering cells resistant to antibacterial compounds [26].

Finally, in vitro experiments demonstrated that specific THC quinoline or AA conjugates have a substantial antimicrobial impact. The findings demonstrated that altering THC’s side chain carbonyl activity improved its antibacterial properties [18,19]. Manjunatha et al. [18] discovered a quinoline derivative with an electron-donating amino group with the highest free radical scavenging activity. These derivatives might have relevant pharmaceutical use and might significantly impact the use of THC in fostering or inhibiting some specific intestinal microbial species in relation to different physiological conditions. In this direction, both THC quinolone and THC amino acidic conjugates might have significant pharmaceutical relevance in the treatment of diseases associated with the change of intestinal microbiota such as diabetes or any other immunologically associated conditions.

## 4. THC Impact upon Diabetes Type II: Relation to the Gut Microbiota and Pancreatic GLP-1

Type II diabetes (T2D) is caused by both hereditary and environmental factors and is characterized by disturbances in glycolipid metabolism. It has been established that dysbiosis of the gut microbiota induced by aging and a high-fat diet is a significant factor that can worsen diabetes [27]. As a result, the intestinal microbiota is seen as a novel therapeutic target for improving diabetes and other metabolic illnesses [28]. Different studies reported that after oral treatment with Cur in people or mice, THC (as one of Cur’s primary metabolites in vivo) could be extracted from the small intestine and the liver [29]. Further, Yuan et al. [30] demonstrated that in diabetic leptin receptor-deficient (*db/db*) mice, THC could enhance blood insulin levels, indicating that THC exhibited a glucose-lowering impact in the early stages of diabetes by boosting compensatory insulin production from the pancreas. Glucagon-like peptide 1 (GLP-1) has been shown to lower fasting blood glucose (FBG) by increasing insulin release from pancreatic cells in a glucose-dependent manner [31]. Furthermore, GLP-1 receptor stimulation promotes cell survival and proliferation [32]. As a result, the hypoglycemic effect found in THC-treated *db/db* mice [30,33] is related to the fact that THC increases the expression of GLP-1 in the pancreas. THC may therefore protect islet cells from persistent hyperglycemia by boosting blood insulin and pancreatic GLP-1 expression.

Over the last two decades, it has been demonstrated that the gut microbiota is critical in controlling obesity and T2D [34,35] and that it can sustain host physiological function by modulating its composition and/or functioning [36,37]. Most bacteria in the gut belong to four recognized phyla: *Firmicutes*, *Bacteroidetes*, *Proteobacteria*, and *Actinobacteria* [38], and the microflora composition is markedly different in *db/db* mice [36]. THC may increase the number of *Bacteroidetes* and *Firmicutes* while decreasing the abundance of *Proteobacteria* and *Actinobacteria* phyla, thus enhancing the variety of the gut microflora. Diabetes is a chronic low-grade inflammatory disease linked with dysregulated gut microbiota [39]. The abnormal proliferation of *Proteobacteria*, in particular, affects gut-resident immune cells, and the production of intestinal mucosal immunoglobulin A (IgA) leads to a compromised intestinal immune system, which is followed by decreased GLP-1 secretion by entero-endocrine cells [40]. *Actinobacteria*, according to Nuli et al. research [41], may comprise bacteria that are favorably connected with lipid metabolism. Authors showed that *Firmicutes* in the colon promote calorie absorption, resulting in obesity [42]. *Bacteroidetes*, which break down soluble fiber into short-chain fatty acids (SCFA) via microbial fermentation, have been linked to the initiation and progression of diabetes [43,44]. Such colonic SCFAs have the potential to be efficient regulators of the plasma peptide YY (PYY), GLP-1, and postprandial insulin levels [45]. Furthermore, SCFAs lower FBG by increasing glucose metabolism indicators [46]. As a result, THC may have a direct regulatory influence on the gut flora, comparable to its parent compound Cur [47]. However, Yuan et al. [30] stressed the importance of significant intestinal microbiota growth as a precondition for the discernible rise in GLP-1 released by the gut, which may explain why the THC-induced glucose-lowering effect in *db/db* mice is detected after 8 weeks of treatment.

**Figure 3:**
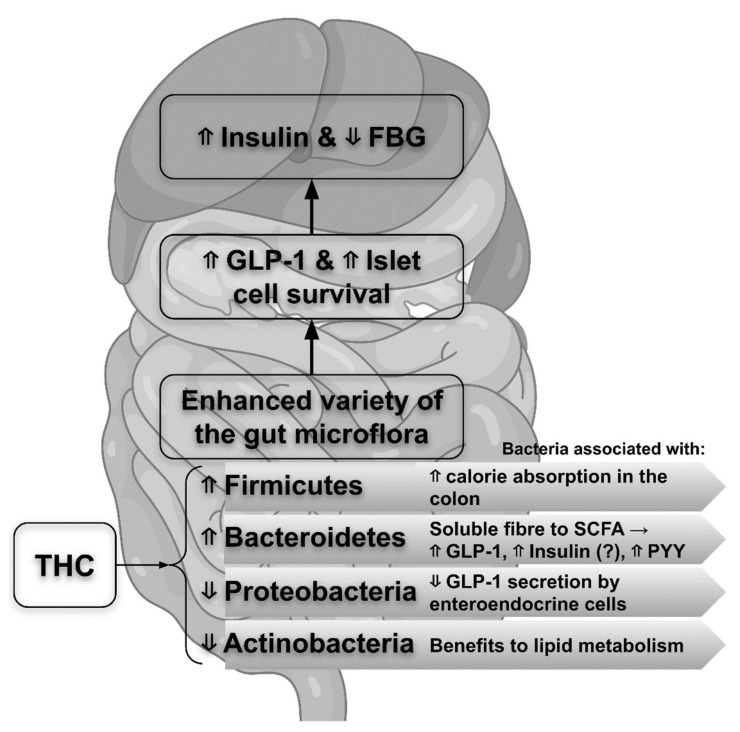
Tetrahydrocurcumin (THC) impact upon diabetes type II: relation to the gut microbiota and pancreatic GLP-1. Data from studies performed on mice. GLP-1—glucagon-like peptide 1; SCFA—short-chain fatty acids; PYY—peptide YY (in blood plasma); FBG—fasting blood glucose; ⇧—stimulatory effect; ⇩—suppressing effect; (?)—conflicting experimental data.

Taking into account the preceding studies, Yuan et al. [30] re-evaluated the link between GLP-1 expression in the pancreas and the four major intestinal microbiota and revealed a positive association between GLP-1 and the abundance of *Bacteroidetes* or *Firmicutes*, indicating that their increased growth could be responsible for the intensity by which THC enhances GLP-1 expression. Meanwhile, a negative connection was discovered between *Proteobacteria* and *Actinobacteria* abundance and GLP-1 expression, demonstrating that THC-induced growth inhibition of these phyla may contribute to GLP-1 upregulation [30]. Overall, it can be inferred that THC can improve gut microbiota dysbiosis, directly lowering FBG levels via modifying GLP-1 expression. However, two different opinions disagree on the direct action of SCFA on pancreatic cells. SCFAs had a positive regulatory impact on insulin release, according to Shah et al. [48], although Orgaard et al. [49] concluded that SCFAs had no physiologically significant effect in perfused mouse pancreas (Figure 3).

In summary, THC demonstrated anti-diabetic properties, which may be connected to its effect on gut microbiota and elevation of GLP-1 expression in the pancreas. The study of Yuan et al. [30] has offered some new aspects about the THC’s hypoglycemic impact associated with regulating the GLP-1 expression. However, further research is needed to determine the relevant processes. Although the mechanisms have not been ultimately proven, at this point, it is worth stressing that THC’s actions upon gut microbiota in conditions of pre-diabetes or T2D could have considerable benefits.

## 5. Anti-Influenza Virus Activity of THC

In their study, Ou et al. [13] examined the anti-type A influenza virus (anti-IAV) bioactivity of numerous curcuminoids in order to elucidate the antiviral mechanism of Cur and to develop stable derivatives with higher biological activity. In this direction, they examined the following questions: (i) Does the stable metabolite THC possess an anti-IAV function? (ii) What is the essential structure responsible for the Cur-mediated anti-IAV activity? It should be noted that Cur can be degraded or bio-transformed very fast in a neutral pH environment and is unstable in physiological conditions [20]. Hence, it is critical to determine whether Cur may exhibit antiviral actions in physiological conditions via its metabolites. THC, as one such metabolite, can cause a decrease in viral production, despite its lesser potency than Cur, indicating that THC may serve as a viable antiviral drug against IAV [13].

The modern pharmaceutical approach declared that it is critical to design antiviral medications that target key components and cellular factors or pathways essential for successful viral replication. The curcuminoids assessed in Ou’s study [13] have been proven to significantly suppress the propagation of the IAV, probably through multiple mechanisms [13]. First, after virus entry, THC dramatically decreased IAV yields, demonstrating that THC likely targets particular phases of the viral replication machinery or dampens cellular signaling involved in the viral replication [46]. Furthermore, the Ras/Mitogen-activated protein kinase (MAPK)/ERK kinase (MEK)/extracellular-signal-regulated kinase (ERK) cascade [50,51,52], the nuclear factor kappa B (NF-κB) pathway [53,54], and the phosphatidylinositol 3-kinase (PI3K)/protein kinase B (PKB) signaling pathways [55,56], are activated and necessary for viral replication. Interestingly, it was proven that THC strongly inhibited the activation of PI3K/PKB and MAPK signaling pathways in human HL-60 leukemia cells [57]. Because THC only mildly decreased viral particle infectivity and did not entirely block viral hemagglutinating activity (HA), it could be expected that modulation of these signaling pathways can contribute significantly to THC-mediated anti-IAV action. In addition, desmethoxycurcumin (DMC) and bisdemethoxycurcumin (BDMC), which have one or no methoxy groups, have the same inhibitory effect as Cur, demonstrating that the methoxy groups are not responsible for the HA [13].

From a chemical point of view, Cur has a symmetrical structure composed of two aromatic rings connected by two unsaturated carbonyl (enone) groups. The enone group serves as the acceptor in the Michael reaction of addition, which is important in intermolecular conjugation with certain proteins [58]. Cur may interact with viral surface proteins and interfere with their function, therefore inactivating virus infectivity via the Michael addition reaction. Hence, in terms of HA inhibition, THC, unlike Cur, failed to reduce viral HA activity [13]. Because each double bond conjugated to the carbonyl moiety of Cur is saturated, the resultant THC cannot function as a Michael reaction acceptor [59]. Furthermore, the presence of glutathione (GSH), one of the most abundant endogenous antioxidants, reduces Cur’s inhibitory effects. This means that incubating Cur with a protein or peptide (e.g., GSH) that has exposed electron-donating functional groups (e.g., the SH group of cysteine in GSH) prevents the Michael acceptor electrophile (MAE) region of Cur from being accessible, thus limiting Cur’s ability to change viral surface proteins [13]. It is crucial to note that GSH does not influence the hemagglutination inhibition (HI) effect. We believe that either the Michael addition reaction is insufficient (other Cur-dependent actions contribute to its anti-IAV effectiveness) or that GSH is not an adequate molecule to compete with the IAV surface protein (i.e., hemagglutinin) for the conjugation to Cur. A variety of phyto-compounds with MAE characteristics failed to suppress IAV’s HA activity, demonstrating that Michael’s addition is not the main contributor to anti-IAV activity [60]. The docking simulation indicated that Cur might create one and two hydrogen bonds with the Asn133 and Gln226 residues on the receptor binding site of the viral HA protein. This explains why GSH competition is ineffective because cysteine is unlikely to be the major residue on the HA protein’s ribosomal binding site (RBS) region that interacts with Cur [60]. Soundararajan et al. [60], employing docking studies, imply that conjugation of Cur with RBS residues on HA reduces the chance of IAV to connect with its cellular receptor, hence preventing viral entrance. This is consistent with the experimental results from Ou et al. [13], showing that incubating viral particles with Cur before cell attachment limits IAV-induced red blood cells agglutination (HI effect), decreases plaque development on Madin–Darby canine kidney (MDCK) cells, and reduces virus production.

Although Cur successfully prevents viral entrance by interacting with the viral HA protein, resistant virus variants would seem to emerge in response to Cur. Nonetheless, Chen et al. [61] reported no IAV variants against Cur, even after five rounds of the blind passage under Cur exposition. This is explained by the fact that Cur-dependent antiviral ability also occurs through acting on cellular factors [61,62,63], and accumulating evidence suggests that antiviral compounds targeting cellular factors appear to be a high barrier to the development of resistant virus variants [54,64].

## 6. THC-Loaded Vaginal Nano-Microbicide for HIV/AIDS Prevention

Microbicides (vaginal/rectal) for the prevention of sexually transmitted human immunodeficiency virus (HIV)/acquired immunodeficiency syndrome (AIDS) infection have received much interest recently [65,66]. Targeting the early stages of HIV, i.e., entrance, fusion, and integration, remains an elusive yet incredibly effective preventive method for HIV infection prevention. Furthermore, clinically accessible entry inhibitors have demonstrated numerous drawbacks in terms of adherence and adoption. As a result, innovative early-stage inhibitors are required to prevent HIV infection caused by sexual transmission. Cur, as a polyphenolic molecule, has shown a wide range of medicinal actions [59,67,68,69,70]. It has also been demonstrated to have significant anti-HIV efficacy as a possible glycoprotein (gp120)-binding inhibitor, protease inhibitor, integrase inhibitor, and topoisomerase II (needed for viral replication) inhibitor [71,72,73]. On the other hand, Cur may cause poor consumer compliance owing to its coloring and odor when applied topically. In light of this, THC, a colorless metabolite of Cur, has been recommended as an alternative to Cur for HIV infection prevention. Yet, no data showing the involvement of THC as an early-stage HIV inhibitor has been revealed. Mirani et al. [14] used computer-assisted in silico molecular docking and molecular dynamic simulation to examine the equivalence of Cur and THC for their gp120-CD4 binding inhibitory activities. The same authors have shown that the THC-gp120 combination has higher stability than the Cur-gp120 complex. Based on in silico data, they predicted that THC is more hydrophobic than Cur, fits deeper in the hydrophobic cavity of the gp120 receptor, and hence inhibits gp120-CD4 binding activity that is equal to or better than Cur’s. As a result, Mirani et al. concluded that THC could be used to manufacture vaginal microbicides to prevent sexually transmitted HIV infection [14].

THC, being a Biopharmaceutics Classification System (BCS) class II molecule, has low solubility, which can lead to poor bioavailability. As a result, Mirani et al. [14] proposed producing THC in an o/w (oil-in-water) microemulsion. Its cytotoxicity in distinct microenvironments (such as cervicovaginal epithelial (CaSki), colorectal epithelial (Caco-2), and peripheral blood mononuclear (PBMC) cells) has been investigated. The obtained cytotoxicity is attributable to the simple glycerol monolaurate (GML)-based o/w microemulsion composition, which likewise exhibited toxicity in CaSki cells and PBMCs [14]. Data utilizing TZM-bl cell lines revealed a four-fold increase in anti-HIV activity of THC-loaded o/w microemulsion compared to THC alone. The increased activity was ascribed not only to improved drug solubility and quick absorption into cells to exert HIV inhibition activity but also to the presence of GML, which possesses intrinsic HIV inhibitory actions. The THC-loaded o/w microemulsion is proposed to be delivered vaginally, so its impact on the vaginal microenvironment (i.e., *Lactobacillus* species, which help to maintain the acidic environment in the vagina by producing lactic acid and H_2_O_2_) was empirically tested to prevent various vaginal infections. GML and THC particularly have not been shown to have any effect on *Lactobacillus* sp. [61]. Taking this into account, the THC-loaded o/w microemulsion was found to be nontoxic at a concentration of 1 mM, which is approximately 1000 times higher than the HIV inhibitory concentration, ensuring the safety of the developed formulation for vaginal delivery.

Due to low retention in the vaginal mucosa, the THC-loaded o/w microemulsion-based system may have poor and short-term effectiveness. To increase retention, Mirani et al. [14] proposed the conversion of the THC-loaded o/w microemulsion into a gel dosage form by using Carbopol Ultrez 10 NF (1% (*w*/*w*)) as a gelling agent. The gel produced in this manner was found to be safe and effective, which opens a new avenue for the application of THC. This is another example of the effective reformulation of THC, which increases its application in the direction of the prevention of sexually transmitted diseases.

## 7. Conclusions

Based on all of the above, it seems crucial to develop new formulations of THC that will increase its bioavailability and efficacy as well as enhance its antibacterial and antiviral properties. For this purpose, in-depth preclinical studies with in vivo, in vitro, and in silico models are needed to evaluate the effects of new THC formulations. Based on the reviewed experimental findings, it seems reasonable to expect that the use of such THC formulations in the prevention and treatment of various ailments related to the microbiotic compartments in the body would be useful and of great importance from a medical perspective.

## Figures and Tables

**Table 1:** Comparison of the structural characteristics and the MIC data of quinoline derivatives and amino acid conjugates of THC against Gram-positive and Gram-negative bacteria. (Modified from Manjunatha et al. [18,19]).

Type of Compound/ conjugate (Figure 2)	Compound	Activity against Bacterial Strain For Each Bacterial Strain the Reported MICs Were Ranked, Highest to Lowest; Longer Bar = Stronger Activity/Lower MIC			
		*B. cereus*	*S. aureus*	*E. coli*	*Y. enterocolitica*
Quinoline derivative	1a	12	1	12	1
	1b	12345	1234	123456	12345
	1c	123456	123456	123453	12345678911
	1d	1	123	1234567	12
	1e	1234	12345	123	1234
	1f	123	12	1234	123
Amino acid conjugate	2a	1234567	1234567891	12345678	1234567
	2b	123456789	123456789	1234567891123	12345678
	2c	1234567891	1234567891123	12345678911	1234567891123
	2d	12345678911	12345678	1234567891	123456789
	2e	12345678	1234567	123456789	123456
	2f	123456789112	123456789112	123456789112	1234567891
	2g	123456789	12345678911	1234567891123	123456789112

## Data Availability

Not applicable.

## Abbreviations

AA	amino acid
AIDS	acquired immunodeficiency syndrome
Anti-IAV	anti-type A influenza virus
BCS	Biopharmaceutics Classification System
BDMC	bisdemethoxycurcumin
BDM-HHC	bis(demethyl)-hexahydrocurcumin
BDM-THC	bis(demethyl)-tetrahydrocurcumin
Caco-2 cells	colorectal epithelial cell lines
CaSki cells	cervicovaginal epithelial cell lines
Cur	curcumin
*db*/*db*	diabetic (leptin receptor-deficient) mouse model
DMC	desmethoxycurcumin
DM-THC	demethyl-tetrahydrocurcumin
ERK	extracellular-signal-regulated kinase
FBG	fasting blood glucose
GLP-1	glucagon-like peptide 1
GML	glycerol monolaurate
gp120	glycoprotein 120
GSH	glutathione
HA	hemagglutinating activity
HI	hemagglutination inhibition
HIV	human immunodeficiency virus
HL-60 cells	human leukemia 60 cells
IAV	type A influenza virus
IgA	immunoglobulin A
MAE	Michael acceptor electrophile
MAPK	mitogen-activated protein kinase
MDCK cells	Madin–Darby canine kidney cells
MEK	mitogen-activated protein kinase
MIC	minimum inhibitory concentration
NF-κB	nuclear factor kappa B
o/w	oil-in-water
PBMCs	peripheral blood mononuclear cells
PI3K	phosphatidylinositol 3-kinase
PKB	protein kinase B
PYY	plasma peptide YY
RBS	ribosomal binding site
SA	sialic acid
SCFA	short-chain fatty acids
SH	sulfhydryl group
THC	tetrahydrocurcumin
TZM-bl	s. JC53-bl (clone 13), JC53BL-13 cells
T2D	type II diabetes
